# Radiation-induced gamma-synuclein in regards to DC function

**DOI:** 10.1038/cddis.2015.253

**Published:** 2015-09-10

**Authors:** J-Y Song, D-S Lim

**Affiliations:** 1 Division of Radiation Cancer Research, Korea Institute of Radiological and Medical Sciences, Seoul 139-706, Korea; 2 Department of Biotechnology, CHA University, Gyeonggi-do 463-400, Korea

Increased γ−synuclein (SNCG) by radiation contributes to immunosuppressive effects via the inhibition of dendritic cell (DC) differentiation and activation, thus making it a potential target for cancer treatment.

Radiotherapy (RT) is a well-established standard tumor treatment, and over half of all cancer patients will receive RT as part of their treatment plan.^1^ Exposure to ionizing radiation (IR) provokes several distinct cell death programs, such as apoptosis, necrosis, mitotic catastrophe, and autophagy, against tumor cells, as well as the surrounding immune cells.^2^ Although RT has traditionally been recognized as cytotoxic and immunosuppressive, in recent years substantial evidence has prompted the re-characterization of radiation as immunomodulatory rather than immunosuppressive. IR-induced ‘danger signals' from dying tumor cells that may contribute to incite a potent anti-tumor immune response via immunogenic cell death (ICD).^3^ However, the interplay between danger signaling patterns behind the trafficking of damage-associated molecular patterns (DAMPs) and their immune-sensing systems appears to be very plastic and highly dependent on the dose and fractionation of radiation, the type of radiation-induced cell death, and the experimental conditions. Thus, whether the effect of intracellular proteins released by RT could be beneficial or detrimental to the final therapy outcome remains controversial.

We have recently demonstrated that single or fractionated doses of radiation induced several secretory proteins in human breast cancer cells.^4^ One of the interesting candidates from the previous study, SNCG, was markedly increased by a high single dose of 10 Gy but not by fractionated irradiation. Several studies have revealed that SNCG is highly expressed in several cancer types, such as the advanced stages of breast, liver, ovarian carcinomas, colon and prostate cancer, and is associated with cancer metastasis and invasiveness.^5^ Therefore, we aim to investigate whether the newly identified secretory SNCG derived from RT-treated dying tumor cells could subsequently elicit anti-tumorigenic immunity or a pro-tumorigenic immune response.

DCs have a vital role as professional antigen-presenting cells that are able to activate naive T cells and initiate T-cell responses, acting as messengers between the innate and adaptive immune systems.^6^ Upon exposure to SNCG, TNF-*α*- or LPS-stimulated semi-mature DCs (smDCs) or mature DCs (mDCs) reduced their expression of several surface molecules such as CD40, CD80, CD86, and MHC-II that contribute to co-stimulation and antigen presentation to T cells. In addition, mDCs in the presence of SNCG significantly reduced the production of the inflammatory cytokines IL-1*β*, IL-6, IL-12, IL-23, IFN-*γ,* and TNF-*α*. Co-culture with SNCG-treated DCs downregulated T-cell proliferation and altered the T-cell cytokine production profile, reducing pro-inflammatory cytokine IFN-*γ* and IL-17 secretion and inducing the anti-inflammatory cytokines IL-4 and TGF-*β*.^7^ Owing to limitations on our ability to evaluate the *in vivo* quantitative and qualitative DC activation in the tumor microenvironment, we further investigated whether the soluble secretory factors from irradiated tumor cells may actually affect DC maturation. Using the Trans-well system, irradiated tumor cells inhibited the activation of LPS-stimulated DCs through a decrease in surface maturation ligands and inflammatory IL-12 and TNF-*α* cytokine production. SNCG derived from RT-treated dying tumor cells may moderate the stimulation of DCs, similar to smDCs, with low expression of phenotypic maturation ligands and the induction of immunosuppressive cytokines, thereby rendering the DCs incapable of efficiently interacting with T cells or eliciting fully immunogenic responses (Figure 1).

Despite the accumulation of emerging evidence, it still remains challenging to understand how, when, and to what extent this dynamic spectrum of DC activation drives tumor-specific anti-tumor immunity, particularly in the context of anti-cancer therapy. In this respect, the pre-existing or therapy-generated tumor microenvironments, as well as the cross-talk between dying cancer cells and DCs, mediated by soluble and vesicular factors, are crucial determinants of the DC maturation status and anti-cancer immune response. Furthermore, the DAMP spectrum can change even for the same cancer cell line depending on the type of treatment; the optimal dosing, timing and sequencing of RT, or other stimuli must be further investigated.

This study indicates that SNCG, which can be released from dying irradiated breast cancer cells, might be at least partially involved in the persistence of tumor resistance against RT, and modulation of SNCG may be a promising approach for anti-cancer therapy. With emerging interest in studying the mechanisms of IR-induced ICD, it is necessary to find novel immunomodulators and analyze certain existing therapies for their potential to cause DC maturation irrespective of whether they induced ICD. This study also cautiously suggests the predictable response of DCs against radiation-induced dying cancer cells.

## Figures and Tables

**Figure 1:**
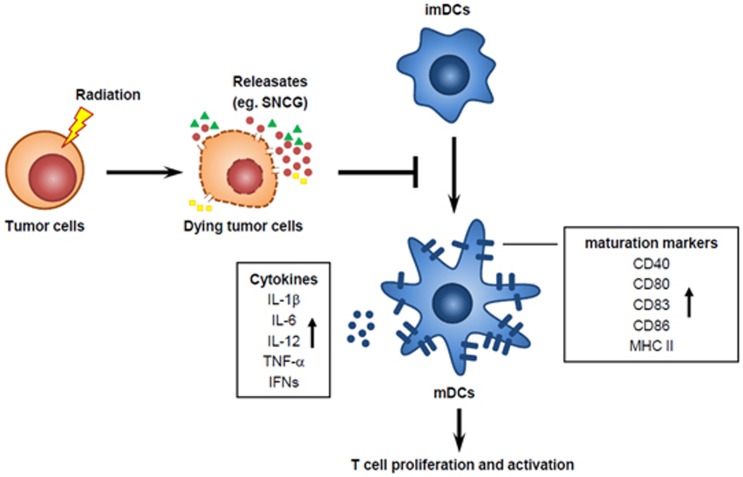
Immunosuppressive effect of SNCG. Tumor cells exposed to radiation undergo different types of tumor cell death such as apoptosis, necrosis, mitotic catastrophe, and senescence. The dying tumor cells emitted various surface molecules and cellular components including already known DMAPs, as well as SNCG. SNCG decreased phenotypic maturation ligands of DCs and downregulated pro-inflammatory cytokine production by DCs, thus led to impede T-cell activation. CD, cluster of differentiation; MHC, major histocompatibility complex; imDCs, immature dendritic cells
